# A Pilot Immunohistochemical Study Identifies Hedgehog Pathway Expression in Sinonasal Adenocarcinoma

**DOI:** 10.3390/ijms25094630

**Published:** 2024-04-24

**Authors:** Matko Leović, Antonija Jakovčević, Ivan Mumlek, Irena Zagorac, Maja Sabol, Dinko Leović

**Affiliations:** 1 Clinical Hospital Center Zagreb, Kišpatićeva 12, 10000 Zagreb, Croatia; matko.leovic@kbc-zagreb.hr; 2 Department of Pathology, Cllinical Hospital Center Zagreb, Kišpatićeva 12, 10000 Zagreb, Croatia; antonia.jakovcevic@kbc-zagreb.hr; 3 Department of Maxillofacial and Oral Surgery, Clinical Hospital Center Osijek, Josipa Huttlera 4, 31000 Osijek, Croatia; mumlek.ivan@kbco.hr; 4 Department of Pathology, Clinical Hospital Center Osijek, Josipa Huttlera 4, 31000 Osijek, Croatia; zagorac.irena@kbco.hr; 5 Laboratory for Hereditary Cancer, Division of Molecular Medicine, Ruđer Bošković Institute, Bijenička Cesta 54, 10000 Zagreb, Croatia; 6 Maxillofacial Surgery Unit, Department of Otorhinolaryngology and Head and Neck Surgery, Clinical Hospital Center Zagreb, Kišpatićeva 12, 10000 Zagreb, Croatia; dinko.leovic@kbc-zagreb.hr

**Keywords:** hedgehog, sinonasal adenocarcinoma, tumor–stroma interaction

## Abstract

Tumors of the head and neck, more specifically the squamous cell carcinoma, often show upregulation of the Hedgehog signaling pathway. However, almost nothing is known about its role in the sinonasal adenocarcinoma, either in intestinal or non-intestinal subtypes. In this work, we have analyzed immunohistochemical staining of six Hedgehog pathway proteins, sonic Hedgehog (SHH), Indian Hedgehog (IHH), Patched1 (PTCH1), Gli family zinc finger 1 (GLI1), Gli family zinc finger 2 (GLI2), and Gli family zinc finger 3 (GLI3), on 21 samples of sinonasal adenocarcinoma and compared them with six colon adenocarcinoma and three salivary gland tumors, as well as with matching healthy tissue, where available. We have detected GLI2 and PTCH1 in the majority of samples and also GLI1 in a subset of samples, while GLI3 and the ligands SHH and IHH were generally not detected. PTCH1 pattern of staining shows an interesting pattern, where healthy samples are mostly positive in the stromal compartment, while the signal shifts to the tumor compartment in tumors. This, taken together with a stronger signal of GLI2 in tumors compared to non-tumor tissues, suggests that the Hedgehog pathway is indeed activated in sinonasal adenocarcinoma. As Hedgehog pathway inhibitors are being tested in combination with other therapies for head and neck squamous cell carcinoma, this could provide a therapeutic option for patients with sinonasal adenocarcinoma as well.

## 1. Introduction

The Hedgehog signaling pathway regulates cell proliferation, differentiation, and tissue polarity during embryonic development. The gradient of Hedgehog ligands is involved in these processes at both short- and long distances [1]. Hedgehog signaling regulates the development of various tissues in the craniofacial region, including teeth, lips, palate, and salivary glands [2,3,4], and its dysregulation during development results in craniofacial deformations, cleft lip and palate, holoprosencephaly, cyclopia, and tooth dysplasia [5]. It is also crucial for the development of epithelial tissues, such as the epidermis, touch domes, hair follicles, sebaceous glands, mammary glands, teeth, nails, and gastric and intestinal epithelium [6]. In adult tissues, its activity is limited to somatic stem cell maintenance and tissue repair [7] and is often upregulated in tumors, where it provides the same signals as during embryogenesis, guiding cell proliferation and differentiation [8].

In humans, three ligands Sonic Hedgehog (SHH), Indian Hedgehog (IHH), and Desert Hedgehog (DHH) act as pathway activation signals in a tissue-specific way. SHH is the most widespread ligand, while DHH is specific for the reproductive system and IHH for bone, cartilage, and digestive tract [9]. The ligands are released from the producing cells, and they can stimulate either the cells that produced them (autocrine activation), the neighboring cells (paracrine activation), or remote tissues if they are distributed long distances by lipid vesicles or as a freely diffusible molecule [10]. This is crucial for the maintenance of the tumor microenvironment, as tumor–stroma interactions can affect tumor cell survival and response/resistance to therapy. The reception of the pathway is regulated by the PTCH1 protein, which is the main receptor for all three Hedgehog proteins. PTCH1 is a tumor suppressor, it keeps control of the Hedgehog pathway through the autoregulative loop, as it is the transcriptional target of the pathway itself. As long as the ligand is present, PTCH1 will translocate from the primary cilia and enable translocation of SMO to the ciliary tip, which will enable activation of GLI transcription factors (GLI1, GLI2, and GLI3). However, when the ligand is not present, PTCH1 will stay localized in the cilia, preventing the accumulation of SMO and therefore preventing the activation of GLI transcription factors [11]. The presence of PTCH1 on the cell membrane signifies that the cell is in a ready state to receive the Hedgehog ligand(s). 

The Hedgehog pathway has been found upregulated in head and neck tumors in both in vitro and in vivo models. We and others have identified overexpression of Hedgehog pathway components in head and neck squamous cell carcinoma (HNSCC) [12,13]. Activation of the pathway in HNSCC has been associated with invasion into the bone [14], lymph node invasion [15], radioresistance [16], and worse overall survival [17]. Squamous cell carcinomas are the most frequent tumor types in the upper aerodigestive tract, but other neoplasms can also be found, such as salivary gland-type tumors and sinonasal adenocarcinoma [18]. Sinonasal adenocarcinomas most frequently occur in the ethmoidal sinus and nasal cavum. According to the recent WHO Classification of Tumors, sinonasal adenocarcinomas are considered as tumors of epithelial origin and are classified into two categories: intestinal type adenocarcinomas (ITACs) and non-intestinal type adenocarcinomas (non-ITACs). ITAC is the second most common type of sinonasal adenocarcinoma (after squamous cell carcinoma), which usually has an aggressive clinical presentation, invading the bone and surrounding tissues: orbit, anterior and middle cranial fossa, etc. Non-ITACs are mostly of low-grade malignant potential, without bone destruction [19]. It is considered that ITAC is developed through the process of intestinal metaplasia of sinonasal olfactory epithelium. ITAC histologically resembles colonic adenocarcinoma and can occur in five histological subtypes: colonic, papillary, solid, mucinous, and mixed. Its development is mostly connected with hard-wood dust exposure and leather manufacturing. The incidence of ITAC among these individuals is 500–1000 times higher compared to the non-exposed population [20,21]. Chronic exposure and irritation by organic dust lead to chronic inflammation, which can stimulate tumor initiation [22,23]. Sinonasal ITAC and “true” intestinal adenocarcinoma share similar immunohistochemical profiles. In both, expressions of CK20, CDX-2, vilin, and MUC2 are present. On the other hand, these markers are not expressed in non-ITACs. Contrary to colorectal carcinoma, *KRAS* and *BRAF* mutations are rare in ITAC. Expression of EGFR protein is stronger in patients with ITAC exposed to wood dust than in those exposed to leather dust. In the subgroup of patients who were not exposed to organic dust, EGFR expression is absent [20,21]. Metastatic spread of intestinal adenocarcinomas to the sinonasal tract is rare [24]. Surgery is the primary method of treatment for these tumors. Data considering survival vary depending on the type of treatment, design of study cohorts, and methodology of adverse events calculation. In the study by Cantu et al., the 5-year and 10-year cause-specific mortality for ITACs was reported as 44% and 53%, respectively [25]. A population-based study of 848 patients with ITAC revealed a 5-year relative survival rate of 63 ± 2.1% [26]. A study of 169 endoscopically treated patients revealed a 5-year overall survival of 68.9% and event-free survival of 63.3% [27]. A recent study of 535 patients, including those treated by radiotherapy and chemotherapy, reports a 5-year overall survival of 52% [28].

In this study, we investigated the expression of six Hedgehog pathway proteins (PTCH1, GLI1, GLI2, GLI3, SHH, and IHH) on 21 formalin-fixed paraffin-embedded (FFPE) samples of sinonasal adenocarcinoma: 18 ITAC and 3 non-ITACs. Six colon adenocarcinoma samples were used as controls to compare to the ITAC subtype. As the Hedgehog signaling pathway is also implicated in intestinal development, homeostasis, and colon adenocarcinoma, and IHH is the Hedgehog ligand relevant for the colon adenoma formation, this ligand was included in addition to the most prevalent SHH [29,30,31]. Additionally, three samples of salivary tumors were used as an outlier subtype of upper aerodigestive tract tumors, where involvement of the Hedgehog pathway has also been demonstrated [32]. To our knowledge, this is the first study to identify Hedgehog pathway upregulation in sinonasal adenocarcinoma.

## 2. Results

**Figure 1:**
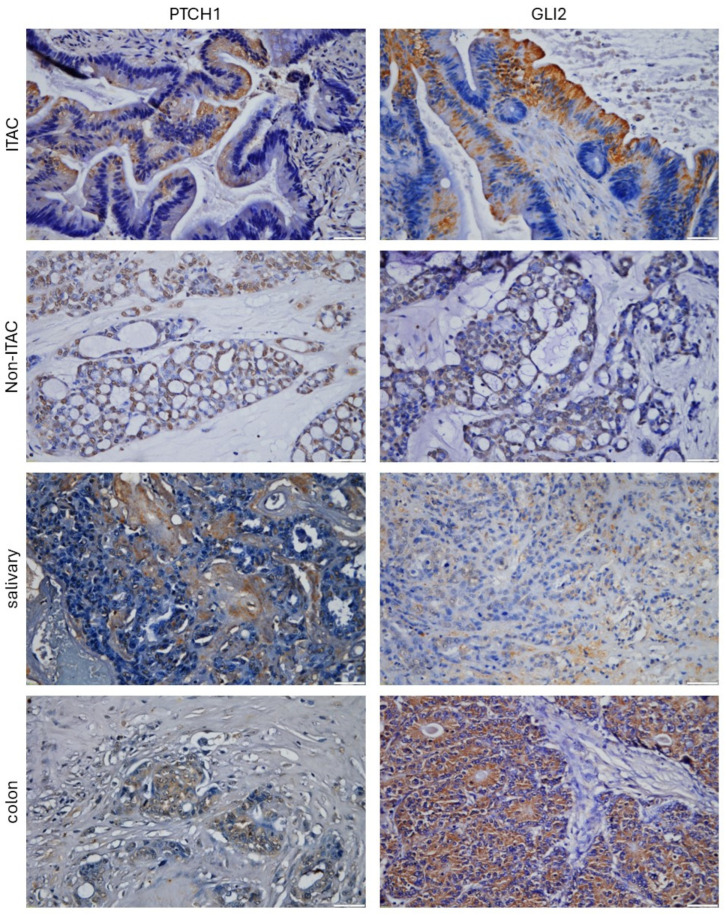
Examples of IHC staining for GLI2 and PTCH1 proteins in ITAC (cytoplasmatic staining in tumor cells), non-ITAC (cytoplasmatic and nuclear staining in tumor cells), salivary (cytoplasmatic staining in stromal cells), and colon (cytoplasmatic staining in tumor cells) adenocarcinomas. Scale bar = 50 µm.

In total, 30 FFPE samples were stained for Hedgehog pathway proteins GLI1, GLI2, GLI3, PTCH1, SHH, and IHH. Immunohistochemical staining showed positive staining of these signaling pathway proteins in sinonasal adenocarcinoma (Figure 1, Table 1).

Overall, the most frequently detected protein was GLI2; it was found in 90% of all tumors, and 66.7% of tumor stromal tissues on average. It was also frequently detected in healthy tissues, 88.3% on average. In all cases, GLI2 staining was detected in the cytoplasm, and some samples also showed some nuclear staining. All three GLI proteins are transcription factors, which can be detected in the cytoplasm when the ligand is not present and in the nucleus when the binding of the ligand activates the signaling cascade. 

**Figure 2:**
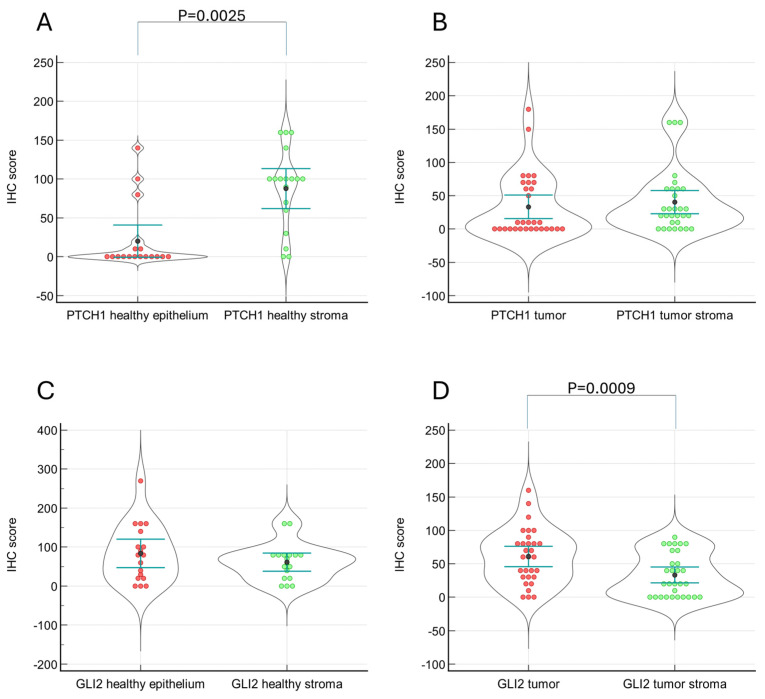
Staining scores of PTCH1 and GLI2 in healthy tissues and tumor tissues for all analyzed samples. PTCH1 staining differs in the healthy tissues, where the stromal compartment shows stronger staining compared to the epithelial compartment (*p* = 0.0001). GLI2 staining differs in the tumor samples, where the tumor tissues show stronger staining compared to the tumor stromal compartment (*p* = 0.0052). (**A**): PTCH1 staining in healthy tissues, (**B**): PTCH1 staining in tumor tissues, (**C**): GLI2 staining in healthy tissues, (**D**): GLI2 staining in tumor tissues.

The second most frequently detected protein was PTCH1, with 53.3% positive tumor samples and 76.6% positive tumor stroma. Interestingly, in the healthy tissues, the stromal compartment was more often positive for PTCH1 than the epithelial compartment (*p* = 0.0025), while this was not the case when comparing the tumor and the tumor stroma compartment (Figure 2). For GLI2 scores, there is no difference between healthy tissues, while for the tumor samples, there is an increase in scores in the tumor tissues compared to the tumor stroma compartment (*p* = 0.0009). Interestingly, in the salivary tumors, the expression pattern of PTCH1 differs from the other analyzed tumor types: Healthy epithelium shows PTCH1 positivity, whereas none of the other tissues showed this pattern. 

Some positivity was also detected for GLI1 protein, 16.7% of tumor samples on average, while other examined proteins of the Hedgehog signaling pathway were mostly undetectable (Table 1). GLI1 was not detected in the colon adenocarcinoma, while some positive samples were detected for the ITAC and non-ITAC and the salivary tumor. The distribution of IHC scores for GLI1, GLI2, and PTCH1 for all samples and all tumor types is presented in Supplementary Figure S1.

**Figure 3:**
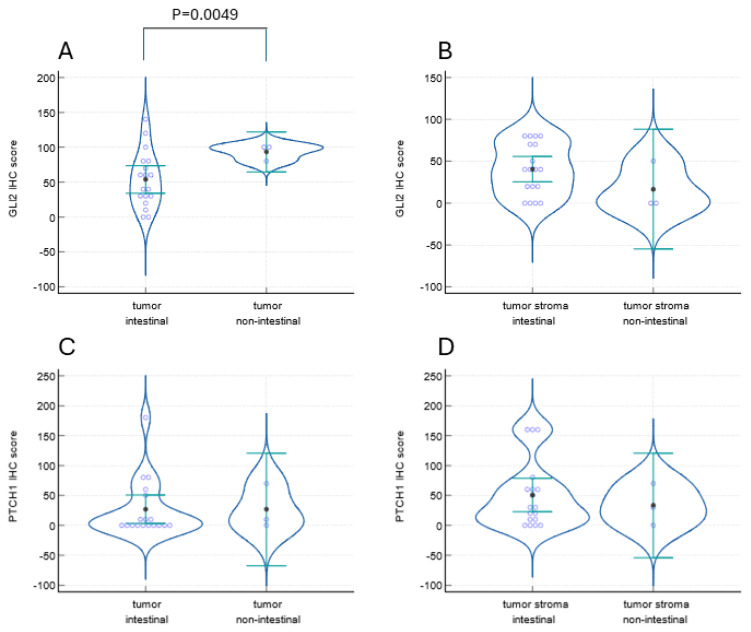
Comparison of the GLI2 and PTCH1 staining scores for the intestinal and non-intestinal subtypes of sinonasal adenocarcinoma. GLI2 score is slightly higher in the non-intestinal tumors compared to the intestinal ones (*p* = 0.0049), while for PTCH1, there are no differences in staining between the two subtypes. (**A**): GLI2 staining in the tumor tissue for ITAC and non-ITAC, (**B**): GLI2 staining in the stroma for ITAC and non-ITAC, (**C**): PTCH1 staining in the tumor tissue for ITAC and non-ITAC, (**D**): PTCH1 staining in the stroma for ITAC and non-ITAC.

The focus of further analyses was on the two most abundant proteins, GLI2 and PTCH1. First, the staining scores of ITAC and non-ITAC subtypes of sinonasal adenocarcinoma were compared. As the non-ITAC subtype is very rare compared to the intestinal subtype, only three samples of this type were collected in our cohort. The staining score of GLI2 in the non-ITAC subtype seems to be higher than the ITAC subtype, even though this may be the consequence of the small number of non-ITAC samples and needs to be verified on a larger cohort (*p* = 0.0049) (Figure 3A). This does not seem to be the case for PTCH1 staining (Figure 3C). There were also no differences between the two subtypes in the stromal compartment for these two proteins (Figure 3B,D). Therefore, in our further analysis, we have grouped the ITAC and non-ITAC subtypes into a single group of sinonasal adenocarcinoma.

When analyzing staining scores of sinonasal adenocarcinoma with the two control groups, the colon adenocarcinoma and the salivary adenocarcinoma, no differences in distribution were found between the three tumor subtypes regarding PTCH1 and GLI2 staining scores (Supplementary Figure S1).

**Figure 4:**
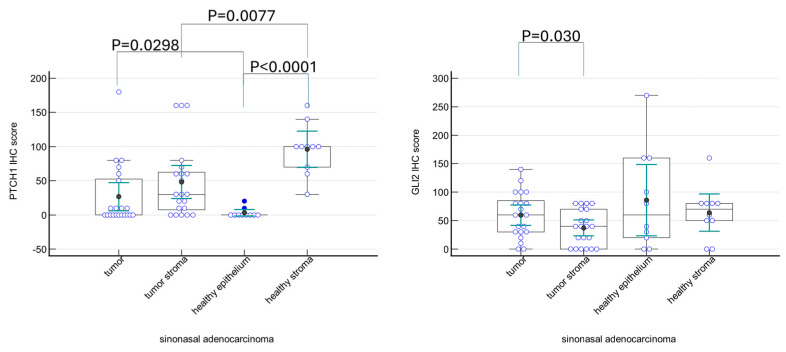
IHC scores for PTCH1 and GLI2 proteins in sinonasal adenocarcinoma. PTCH1 expression is the highest in the healthy stromal cells, and it is downregulated in the tumor stroma (*p* = 0.0077). On the other hand, healthy epithelium shows very weak or no expression of PTCH1, which is increased in tumor tissue (*p* = 0.0298). GLI2 expression is mostly uniform throughout the groups, with stronger staining of tumor tissue compared to its stromal compartment.

The involvement of the tumor stroma compartment in tumor biology was further examined on the sinonasal adenocarcinoma subgroup, as other groups were used as referent groups, and had too few samples for a meaningful statistical analysis. The trend seen in all samples is again seen here in the sinonasal adenocarcinoma subgroup, with PTCH1 expression significantly different between the healthy epithelium and healthy stroma (*p* < 0.0001), and GLI2 expression significantly different between the tumor and tumor stroma (*p* = 0.030) (Figure 4).

The absence of either ligand suggests that the pathway is not activated by an autocrine mechanism in these tumor samples. Rather, the presence of PTCH1 and GLI proteins may signify a ready state to receive the ligand signal from remote tissues. Alternatively, it may suggest that their expression is the result of non-canonical activation of the pathway resulting in the expression of the two known downstream targets, PTCH1 and GLI2. 

Based on the analysis of the different sample regions, it can be deduced that the PTCH1 protein is preferentially expressed in the stromal compartment of both sinonasal and referent colon adenocarcinoma. This supports the paracrine signaling model in these tumor types, where the stromal cells express HH-GLI pathway receptors and activate downstream signaling, while the activation signal does not originate in the stroma but rather in the tumor (not the case here) or in remote tissues. It is likely that the stromal cells can, upon reception of the signal, activate the pathway and produce various growth factors and cytokines that can support tumor growth. This analysis, however, is beyond the scope of this work.

## 3. Discussion

As the Hedgehog signaling pathway regulates the development of the craniofacial structures and epithelium, it is not surprising that its aberrant activation can be detected in tumors developing from these tissues. The involvement of Hedgehog signaling in squamous cell carcinoma of the head and neck region has been well-documented [33]. The Hedgehog signaling pathway has been implicated in salivary gland neoplasms. Vidal et al. have demonstrated positive SHH and GLI1 staining of salivary glands and their neoplasms [32]. In our study, we did not detect SHH in the salivary glands or salivary tumors, but we did detect both GLI2 and PTCH1, which Vidal et al. did not include in their study, confirming the activation of the Hedgehog pathway in the salivary gland tumors. Olfactory neuroblastoma (ONB), another rare tumor of the epithelium in the nasal cavity, also showed activation of the Hedgehog pathway (IHC staining of PTCH1, GLI1, and GLI2), as well as inhibition by Hedgehog pathway inhibitor cyclopamine in vitro on two ONB cell lines [34]. However, there is no information on sinonasal adenocarcinoma. As sinonasal adenocarcinoma is most frequently of the ITAC subtype, which resembles colorectal tumors, we included six colon adenocarcinoma samples to compare to the ITAC.

The activation of the Hedgehog pathway in colon adenocarcinoma epithelial cells has been demonstrated 15 years ago [35]. At the same time, it has been demonstrated that Hedgehog signaling is also involved in the differentiation and renewal of colon epithelial lining as PTCH1, GLI1, and GLI2 were detected in the crypts [36]. In our study, the results were similar, with GLI2 detected in both healthy colon and tumors. However, Alinger et al. also detected SHH and DHH expression in the epithelium of their samples, while we did not. As the Hedgehog pathway is often activated in wound healing and tissues that require constant renewal, such as colon and oral epithelium, it is not surprising to detect its expression in healthy tissues [37]. Another possible explanation for the presence of pathway proteins is the sampling of the control tissues, which were taken from the same individual, and may therefore be affected by unseen processes during field cancerization, which is very common in tumors of the head and neck [38]. Mazumdar et al. have tested two Hedgehog pathway inhibitors on colon cancer cell lines and concluded that inhibition works better at the level of GLI proteins rather than at the level of membrane component SMO, suggesting that GLI activation, possibly through non-canonical pathways, is the contributing factor in colon carcinogenesis [39].

Tumor–stroma communication is extremely important in the maintenance of the favorable microenvironment for cancer progression. For example, in the healthy intestine, the ligand IHH is produced in the epithelial cells and received by the mesenchymal cells [29]. In the mouse model, downstream components of the signaling pathway are confined to the stromal compartment. Furthermore, the same study showed that the expression of downstream components GLI1, GLI2, and GLI3 is disconnected from the expression of the ligands and receptors. Activation of Hedgehog signaling in the stromal compartment resulted in the induction of epithelial differentiation markers and restriction of colonic stem cell markers [40]. In oral squamous cell carcinoma, SHH and GLI1 are both found in the stromal compartment and could be the source of the ligand for both paracrine and autocrine activation in the tumor cells [41]. In fact, SHH can contribute to therapeutic resistance in HNSCC and is a predictor of shorter overall survival and disease-free survival in patients treated with cisplatin [42]. Hypopharyngeal squamous cell carcinoma cell line FaDu can be inhibited by Hedgehog inhibitor JK184 when cells are implanted into the maxillary sinus of nude mice [43]. This, however, does not reflect the potential biology of the sinonasal cavity, but rather the HNSCC cell line itself, and it has been demonstrated that these cells respond to Hedgehog pathway inhibition [44].

When comparing the literature data and the staining patterns of sinonasal adenocarcinoma in our study to the colon and HNSCC tumors, sinonasal adenocarcinoma shows more similarities to the colon than to the HNSCC, primarily due to the lack of co-localization of the upstream and downstream components of the signaling pathway. This makes Hedgehog signaling pathway activation in the sinonasal adenocarcinoma more similar to the paracrine model seen in colon adenocarcinoma than to the autocrine or mixed autocrine plus paracrine model seen in HNSCC. What is especially interesting is the shift of PTCH1 expression, which changes from the healthy stroma to the tumor compartment, and this is matched with the stronger GLI2 staining in the tumor as well. This may indicate that the tumor cells become the receiving cells for the outside signal, resulting in their proliferation.

Sinonasal adenocarcinoma, when inoperable, is often subjected to radiotherapy as part of the treatment protocol. However, radiotherapy can result in unforeseen effects. When HNSCCs are irradiated, there is the activation of GLI1 in the stroma, which contributes to the repopulation of the tumor after therapy and radioresistance [16]. The same is true for colon cancer cells, where SHH and GLI1 expression are increased after irradiation and contribute to tumor repopulation after radiotherapy [45]. Hedgehog pathway inhibitor vismodegib can sensitize HNSCC cell lines to radiation therapy [46]. In the salivary gland, irradiation induces cellular senescence, leading to impaired salivary gland function and dry mouth. It has been demonstrated in both mouse and pig models that the re-introduction of SHH can preserve salivary gland function [47,48]. Therefore, an investigation into Hedgehog pathway expression pre- and post-irradiation would be very informative for these tumors, and it may reflect on patient quality of life and survival. This should be investigated in a separate study with a larger cohort.

The GLI transcription factors were in most cases detected in the cytoplasm of the cells, with occasional nuclear staining, which may signify that the pathway is not activated, but rather poised and ready for activation. This is supported by the fact that ligands required to activate the pathway (SHH and IHH) were not detected in our samples; therefore, there is no signal for translocation of the transcription factors to the nucleus. It is surprising that the Hedgehog ligands, SHH and IHH, have not been detected in our samples. The question remains whether SHH ligand can be delivered from a remote tissue, and if such SHH-expressing cells exist in this region. According to some recent studies, SHH can be detected in the nasal mucus and is decreased in patients with hyposmia [49]. The same was demonstrated by the same group for the parotid saliva and patients with taste dysfunction [50]. Maurya et al. hypothesize that the SHH protein present in the nasal mucus is necessary for the activation of the Hedgehog pathway in the olfactory cilia, as they have demonstrated that the Hedgehog pathway is required for olfactory perception in a mouse model [51]. Therefore, it is possible that this can also be the source of the ligands for the sinonasal adenocarcinoma, but this needs to be investigated further.

## 4. Materials and Methods

In this pilot retrospective study, archival samples of 18 intestinal adenocarcinoma of the sinus, 3 non-intestinal adenocarcinoma of the sinus, 3 salivary gland tumors, and 6 colon adenocarcinoma FFPE samples were collected from the Department of Otorynolaryngology and Head and Neck Surgery, Clinical Hospital Centre Zagreb and the Department of Oral and Maxillofacial Surgery, Clinical Hospital Osijek. For 18 samples, accompanying healthy tissue controls from the same patient were also available. Ethical approval was granted by the Ethical Committee of the Clinical Hospital Centre Zagreb (no. 02/21 AG) on 25 November 2019. Due to the retrospective nature of the study, patient consent was not required.

### 4.1. Immunohistochemical Staining

FFPE slides (thickness 4–5 µm) were deparaffinized in Bioclear (Biognost, Zagreb, Croatia), rehydrated in 100%, 90%, and 70% ethanol, and finally in water. Rehydrated slides were warmed to 100 °C in citrate Target retrieval solution pH 6 (Agilent, Santa Clara, CA, USA) and left to cool to room temperature before proceeding with blocking of the endogenous peroxidase activity by 3% hydrogen peroxide in methanol (Kemika, Zagreb, Croatia). Slides were washed in TBST buffer and blocked with serum-free Protein block (Agilent, Santa Clara, CA, USA), followed by incubation with the following antibodies overnight at 4 °C: anti-PTCH1 (1:100, 1750-1-AP, ProteinTech, Planegg-Martinsried, Germany), anti-GLI1 (1:100, NB600-600, Novus Biologicals, Centennial, CO, USA), anti-GLI2 (1:100, sc-271786, Santa Cruz Biotechnology, Dallas, TX, USA), anti-GLI3 (1:200, GTX104362, GeneTex, Irvine, CA, USA), anti-SHH (1:100, sc-365112, Santa Cruz Biotechnology, Dallas, TX, USA), and anti-IHH (1:100, sc-271101, Santa Cruz Biotechnology, Dallas, TX, USA). For negative control, the primary antibody was replaced with 2%BSA/TBST. Following incubation, the detection was performed using the LSAB2 universal kit (Agilent, Santa Clara, CA, USA), the signal was visualized with DAB (Agilent, Santa Clara, CA, USA), and the slides were counterstained with hematoxylin (Biognost, Zagreb, Croatia). Slides were dehydrated using 70%, 90%, and 100% ethanol solutions and Bioclear and fixed in the Biomount medium (Biognost, Zagreb, Croatia). 

### 4.2. Slide Analysis

Slides were examined by an expert pathologist and scored by assessing the signal intensity (0–3) and percentage of positive cells (0–100%) for both the tumor mass and the tumor stroma. Additionally, where available, the accompanying healthy tissues were scored in the same way, examining the healthy epithelium and healthy stroma. Final scores were generated by multiplying the staining intensity with the percentage of positive cells for each of the four examined regions separately. Images were taken using the Olympus BX51 microscope with OLYMPUS stream Essentials 2.4 licensed software. The data were analyzed in MedCalc for Windows (v.22-021) using the Kruskal–Wallis test for multiple group comparison, paired samples *t*-test for comparisons between scores between two regions within the sample, and independent samples *t*-test for comparisons of scores between tumor types.

## 5. Conclusions

Based on our preliminary results on a small cohort of sinonasal adenocarcinoma samples, we conclude that Hedgehog pathway activation can be detected in these samples. The most frequently detected proteins were PTCH1, the pathway receptor, and GLI2, which seems to be the dominant pathway activator among the three GLI proteins. GLI1 expression can also be detected in a smaller fraction of samples. No expression of ligands, SHH and IHH, was detected, suggesting that the ligands are not produced in these tissues but rather delivered from remote tissues, possibly the nasal mucus. The signal detected in the stromal compartment suggests that the mode of Hedgehog signal transduction is paracrine in sinonasal adenocarcinoma. Based on these findings, the sinonasal adenocarcinoma shows more similarities to the colon adenocarcinoma than the HNSCC or salivary gland tumors, which concurs with analyses of other markers by other authors. This may be relevant for the development of future therapies, as upstream inhibitors of Hedgehog signaling might be less effective than those targeting downstream components.

## Figures and Tables

**Table 1:** Summary table of the number and percentage of samples with positive staining for the HH-GLI pathway proteins GLI1, GLI2, GLI3, PTCH1, SHH, and IHH in four tumor types (colon adenocarcinoma, sinonasal adenocarcinoma of the intestinal type, sinonasal adenocarcinoma of the non-intestinal type, and salivary adenocarcinoma) for four different regions (tumor mass, tumor stroma, healthy epithelium, and healthy stroma).

**Protein**	**Tumor Regions**	**Total n**	**All**	**Colon (n = 6)**	**Intestinal (n = 18)**	**Non-Intestinal (n = 3)**	**Salivary (n = 3)**
GLI1	Tumor	30	5 (16.7%)	0 (0%)	4 (22.2%)	1 (33.3%)	0 (0%)
	Tumor stroma	30	5 (16.7%)	0 (0%)	4 (22.2%)	1 (33.3%)	0 (0%)
GLI2	Tumor	30	27 (90%)	5 (83.3%)	16 (88.9%)	3 (100%)	3 (100%)
	Tumor stroma	30	20 (66.7%)	4 (66.7%)	14 (77.8%)	1 (33.3%)	1 (33.3%)
GLI3	Tumor	30	1 (3.3%)	0 (0%)	1 (5.5%)	0 (0%)	0 (0%)
	Tumor stroma	30	1 (3.3%)	0 (0%)	0 (0%)	0 (0%)	1 (33.3%)
PTCH1	Tumor	30	16 (53.3%)	4 (66.7%)	8 (44.4%)	2 (66.7%)	2 (66.7%)
	Tumor stroma	30	23 (76.7%)	5 (83.3%)	14 (77.8%)	2 (66.7%)	2 (66.7%)
SHH	Tumor	30	0 (0%)	0 (0%)	0 (0%)	0 (0%)	0 (0%)
	Tumor stroma	30	0 (0%)	0 (0%)	0 (0%)	0 (0%)	0 (0%)
IHH	Tumor	30	0 (0%)	0 (0%)	0 (0%)	0 (0%)	0 (0%)
	Tumor stroma	30	0 (0%)	0 (0%)	0 (0%)	0 (0%)	0 (0%)
	**Healthy Regions**	**Total n**	**All**	**Colon (n = 6)**	**Intestinal (n = 9)**	**Non-Intestinal (n = 1)**	**Salivary (n = 2)**
GLI1	Healthy epithelium	18	1 (5.5%)	0 (0%)	0 (0%)	0 (0%)	1 (50%)
	Healthy stroma	18	2 (11.4%)	0 (0%)	2 (22.2%)	0 (0%)	0 (0%)
GLI2	Healthy epithelium	18	15 (83.3%)	6 (100%)	7 (77.8%)	1 (100%)	1 (50%)
	Healthy stroma	18	15 (83.3%)	6 (100%)	7 (77.8%)	1 (100%)	1 (50%)
GLI3	Healthy epithelium	18	1 (5.5%)	0 (0%)	0 (0%)	0 (0%)	1 (50%)
	Healthy stroma	18	1 (5.5%)	0 (0%)	1 (11.1%)	0 (0%)	0 (0%)
PTCH1	Healthy epithelium	18	6 (33.3%)	2 (33.3%)	2 (22.2%)	0 (0%)	2 (100%)
	Healthy stroma	18	16 (88.9%)	5 (83.3%)	9 (100%)	1 (100%)	1 (50%)
SHH	Healthy epithelium	18	3 (16.7%)	0 (0%)	2 (22.2%)	0 (0%)	1 (50%)
	Healthy stroma	18	3 (16.7%)	0 (0%)	2 (22.2%)	0 (0%)	1 (50%)
IHH	Healthy epithelium	18	0 (0%)	0 (0%)	0 (0%)	0 (0%)	0 (0%)
	Healthy stroma	18	0 (0%)	0 (0%)	0 (0%)	0 (0%)	0 (0%)

## Data Availability

Data are contained within the article and Supplementary Materials.

## Supplementary Materials

The following supporting information can be downloaded at https://www.mdpi.com/article/10.3390/ijms25094630/s1.
