# Supplementary figures and images for: A Pilot Immunohistochemical Study Identifies Hedgehog Pathway Expression in Sinonasal Adenocarcinoma
Source: Int J Mol Sci. 2024 Apr 24;25(9):4630. doi: 10.3390/ijms25094630 (PMC11083810; doi:10.3390/ijms25094630)

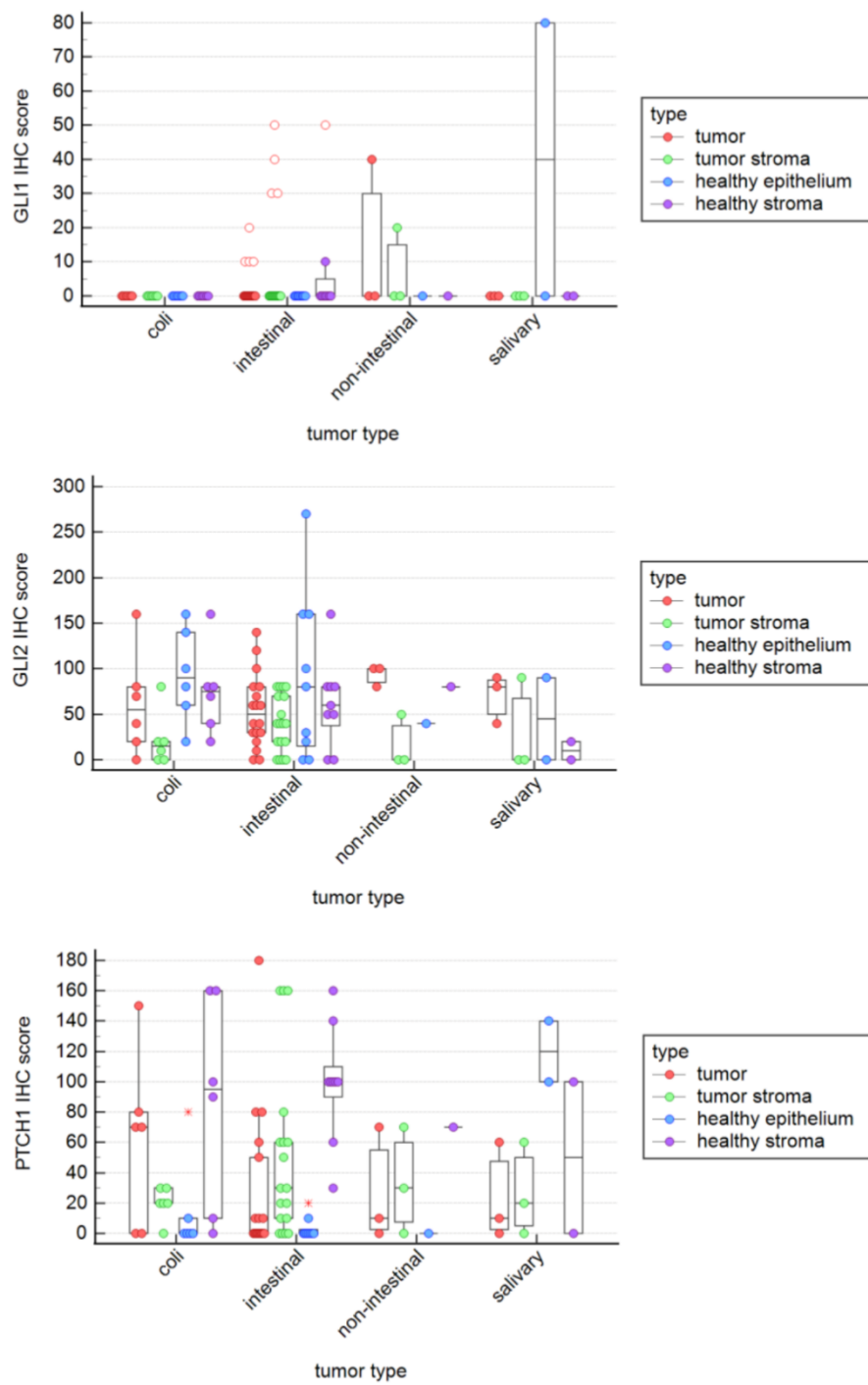

**Figure S1.** GLI1, GLI2 and PTCH1 staining scores in all analyzed samples.

Supplement: Supplementary file 1 [file ijms-25-04630-s001.zip › ijms-2952975-supplementary.pdf]
